# Author Correction: Impacts of convection, chemistry, and forest clearing on biogenic volatile organic compounds over the Amazon

**DOI:** 10.1038/s41467-025-64764-6

**Published:** 2025-10-01

**Authors:** Nidhi Tripathi, Bianca E. Krumm, Achim Edtbauer, Akima Ringsdorf, Nijing Wang, Matthias Kohl, Ryan Vella, Luiz A. T. Machado, Andrea Pozzer, Jos Lelieveld, Jonathan Williams

**Affiliations:** 1 Department of Atmospheric Chemistry, Max Planck Institute for Chemistry, Mainz, Germany; 2 Institute for Atmospheric Physics, Johannes Gutenberg University Mainz, Mainz, Germany; 3 Institute of Physics, University of Sao Paulo, Sao Paulo, Brazil; 4 Climate and Atmosphere Research Center, The Cyprus Institute, Nicosia, Cyprus

Correction to: *Nature Communications* 10.1038/s41467-025-59953-2, published online 20 May 2025

In the version of the article initially published, in the last paragraph of the “BVOCs sensitivity analysis in the Amazonian atmosphere” section, the sentence “… a strong net increase in radiative cooling forcing through O_3_ and aerosols” should have read “… a strong net increase in radiative forcing through O_3_ and aerosols” and has now been corrected in the HTML and PDF versions of the article.

