# Supplementary material for: Impacts of convection, chemistry, and forest clearing on biogenic volatile organic compounds over the Amazon
Source: Nat Commun. 2025 May 20;16:4692. doi: 10.1038/s41467-025-59953-2 (PMC12092753; doi:10.1038/s41467-025-59953-2)
Supplement: Supplementary file 1 — Supplementary Information [file 41467_2025_59953_MOESM1_ESM.docx]

**Impacts of convection, chemistry, and forest clearing on biogenic volatile organic compounds over the Amazon**

N. Tripathi^1*^, B. E. Krumm^1^, A. Edtbauer^1^, A. Ringsdorf^1^, N. Wang^1^, M. Kohl^1^, R. Vella^1,2^, Luiz A. T. Machado^3^, A. Pozzer^1,4^, J. Lelieveld^1,4^, J. Williams^1,4*^

^1^Department of Atmospheric Chemistry, Max Planck Institute for Chemistry, Mainz, Germany

^2^Institute for Atmospheric Physics, Johannes Gutenberg University Mainz, Mainz, Germany

^3^Institute of Physics, University of Sao Paulo, Sao Paulo, Brazil

^4^Climate and Atmosphere Research Center, The Cyprus Institute, 1645 Nicosia, Cyprus.

^*^Corresponding author J. Williams ([Jonathan.Williams@mpic.de](mailto:Jonathan.Williams@mpic.de)) and N. Tripathi ([n.tripathi@mpic.de](mailto:n.tripathi@mpic.de))

**Figures and Text**


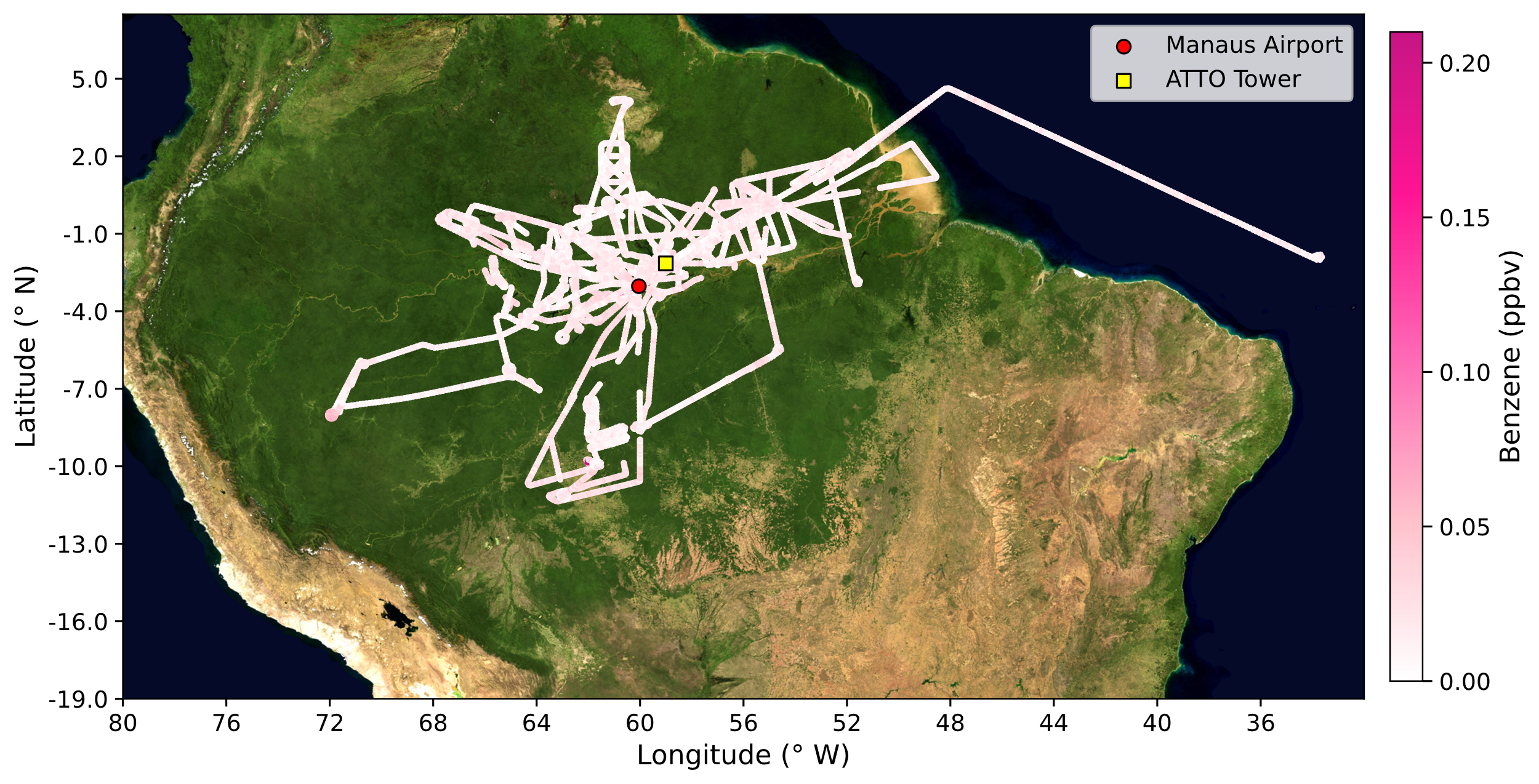


**Supplementary Figure 1.** Spatial distribution of benzene over the Amazonia region. The flight tracks during the CAFE-Brazil campaign over the Amazon rainforest color-coded by benzene mixing ratios. Satellite picture data in this figure obtained from [https://wvs.earthdata.nasa.gov](https://wvs.earthdata.nasa.gov/), NASA Worldview Snapshots.

**
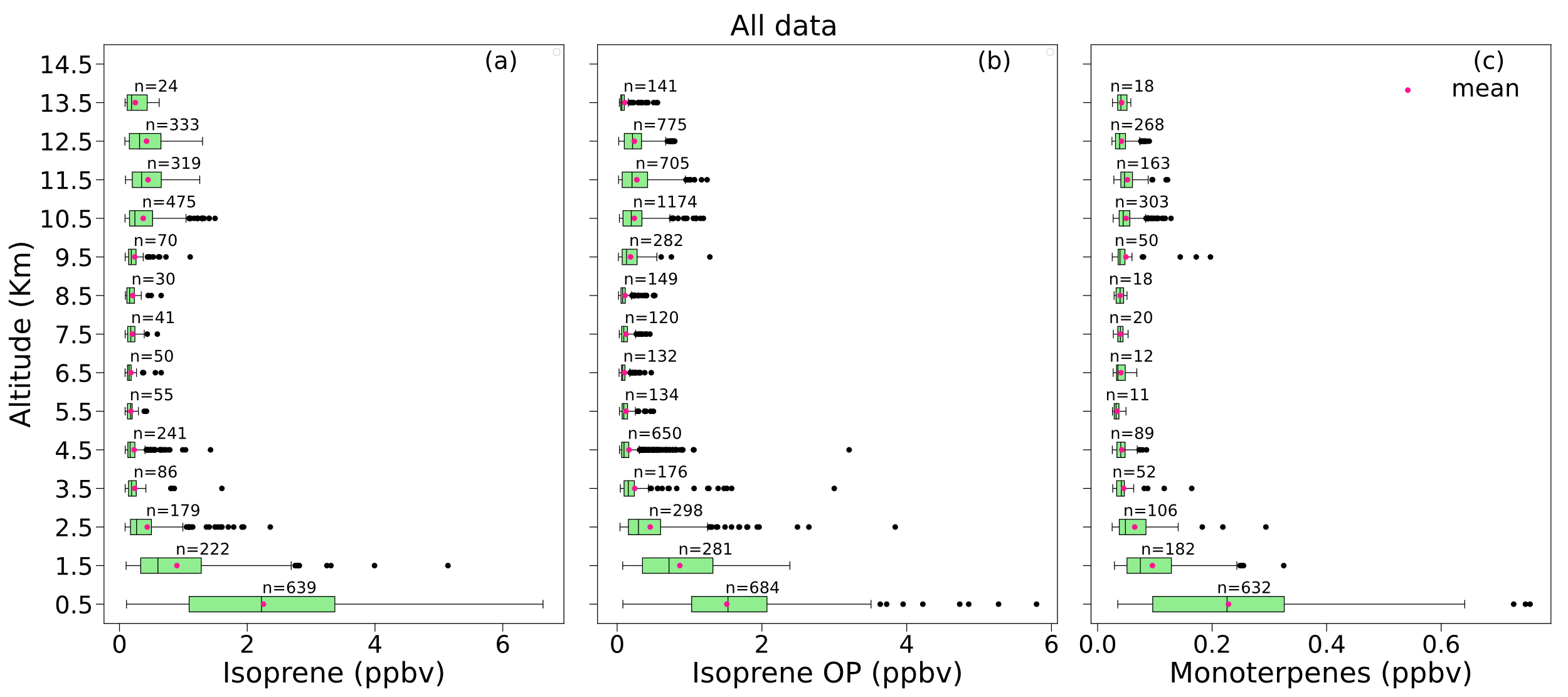
**

**Supplementary Figure 2.** Vertical profiles of a) isoprene, b) isoprene oxidation products (OP), and c) monoterpenes during the CAFE-Brazil campaign over the Amazon rainforest (15 days of data=~125hr). The profiles are binned to a 1 km vertical resolution grid, with the number of data points used for each box displayed above it. The boxes represent the 25th–75th percentiles, with black lines indicating the median while red dots indicate the mean values. Whiskers extend to the lowest and highest data points within 1.5× Interquartile Range (IQR), the data point beyond this range is considered outlier (black dots). Only data above the limit of detection (LOD) is used for this box-whisker plot. The number of data points for these biogenic volatile organic compounds (BVOCs) differs due to their respective LOD values.


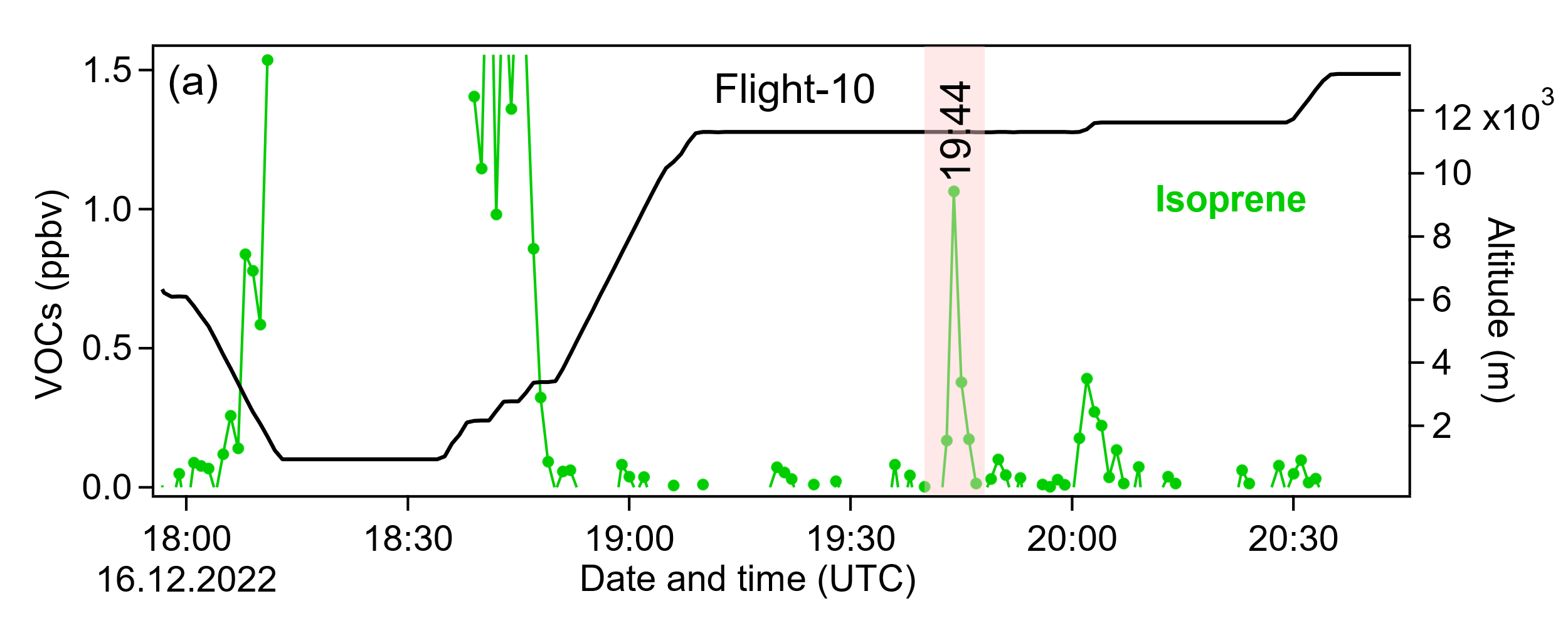


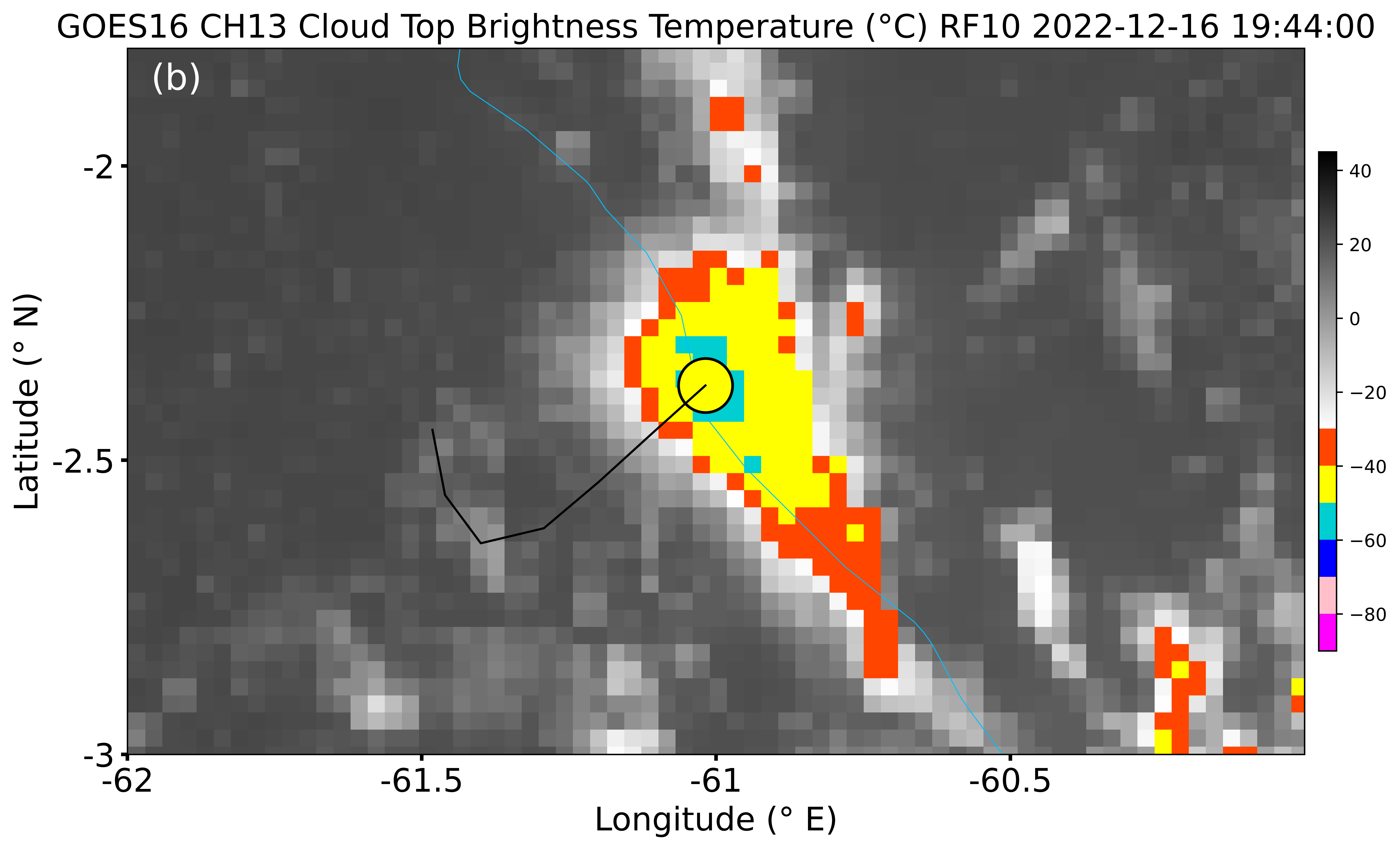


**Supplementary Figure 3.** Isoprene time series and cloud top brightness temperature spatial distribution at 19:40 UTC during Flight-10. a) Time series of isoprene mixing ratio observed during flight 10, with the transparent strip highlighting periods of elevated isoprene levels associated with deep convection. b) The cloud-top brightness temperature, representing the deep convective system during flight 10. Infrared satellite image (GOES-16, band 13: 10.3 µm; <https://ftp.cptec.inpe.br/goes/goes16/retangular/ch13/2022/12/>) indicating the approximate cloud-top brightness temperatures at 19:40 UTC. Temperatures below −40 °C are colored. The yellow circle indicates the HALO aircraft location at 19:44 UTC (15:44 local time), and the colors correspond to the temperatures measured during the flight. The map in this figure was made with Natural Earth. Free vector and raster map data @ naturalearthdata.com.


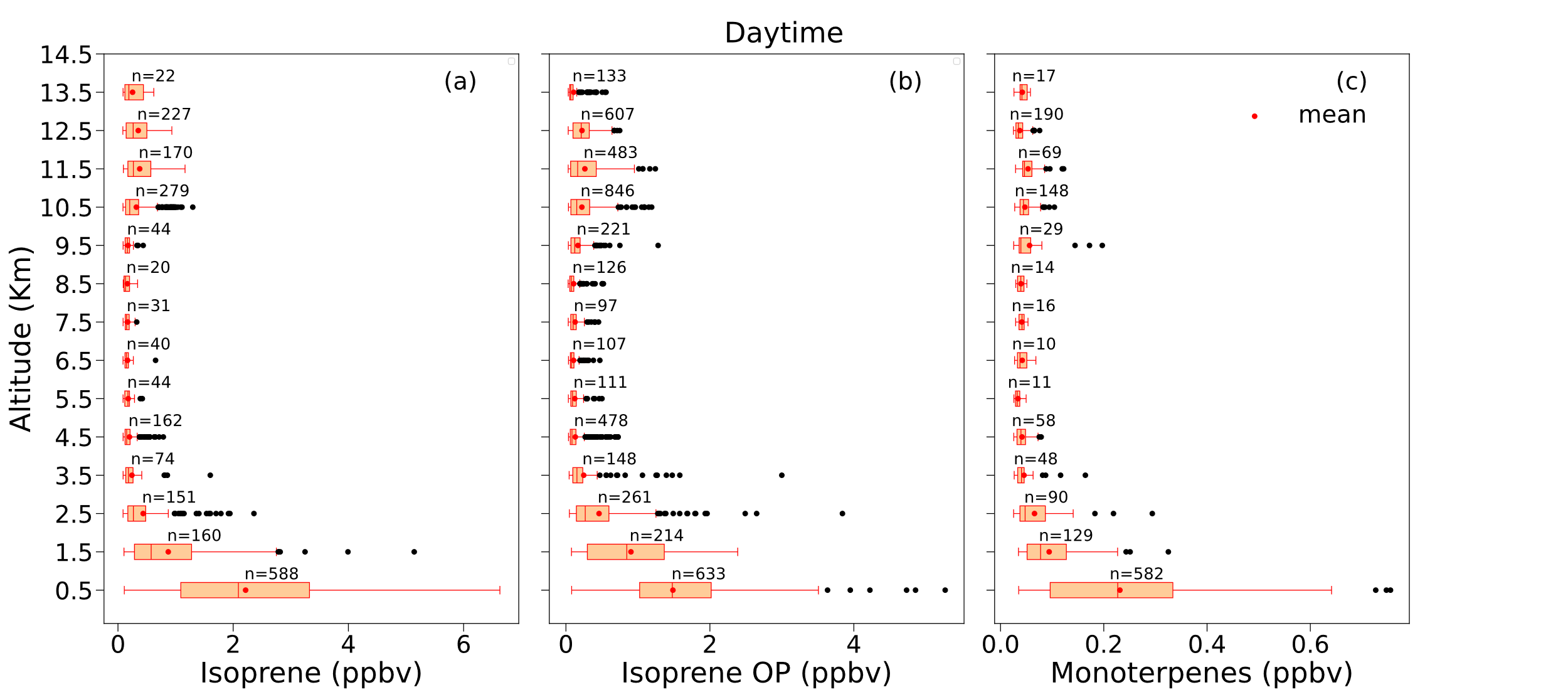


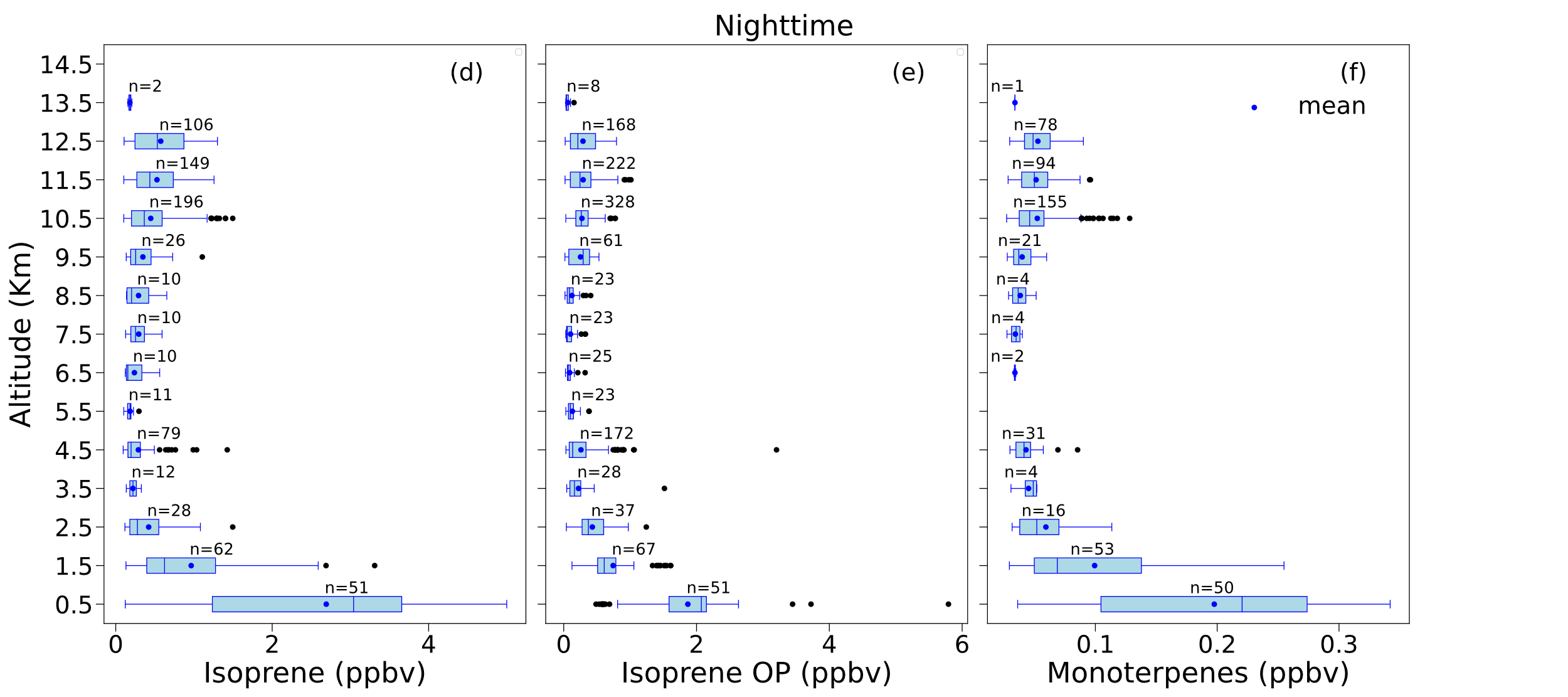


**Supplementary Figure 4.** Vertical profiles of isoprene, its oxidation products, and monoterpenes during the CAFE-Brazil campaign over the Amazon rainforest during the day (upper panel, a-c) and night (lower panel, d-f). Panels a) and d), correspond to isoprene; b) and e) to its oxidation products; and c) and f) to monoterpenes. The profiles are binned to a 1 km vertical resolution grid, with the number of data points used for each box displayed above it. The boxes represent the 25th–75th percentiles, with red (daytime)/blue (nighttime) lines indicating the median while dots indicate the mean values. Whiskers extend to the lowest and highest data points within 1.5× Interquartile Range (IQR), the data point beyond this range is considered outlier. Black dots represent outliers. A total of 15 days of flight (~125 hr) data was used, with daytime data spanning 06:00 to 18:00 local time (~100 hr) and nighttime data (~25 hr) covering 18:00 to 06:00 local time. Only data above the limit of detection (LOD) is used for this box-whisker plot. The number of data points for these biogenic volatile organic compounds (BVOCs) differs due to their respective LOD values.

**
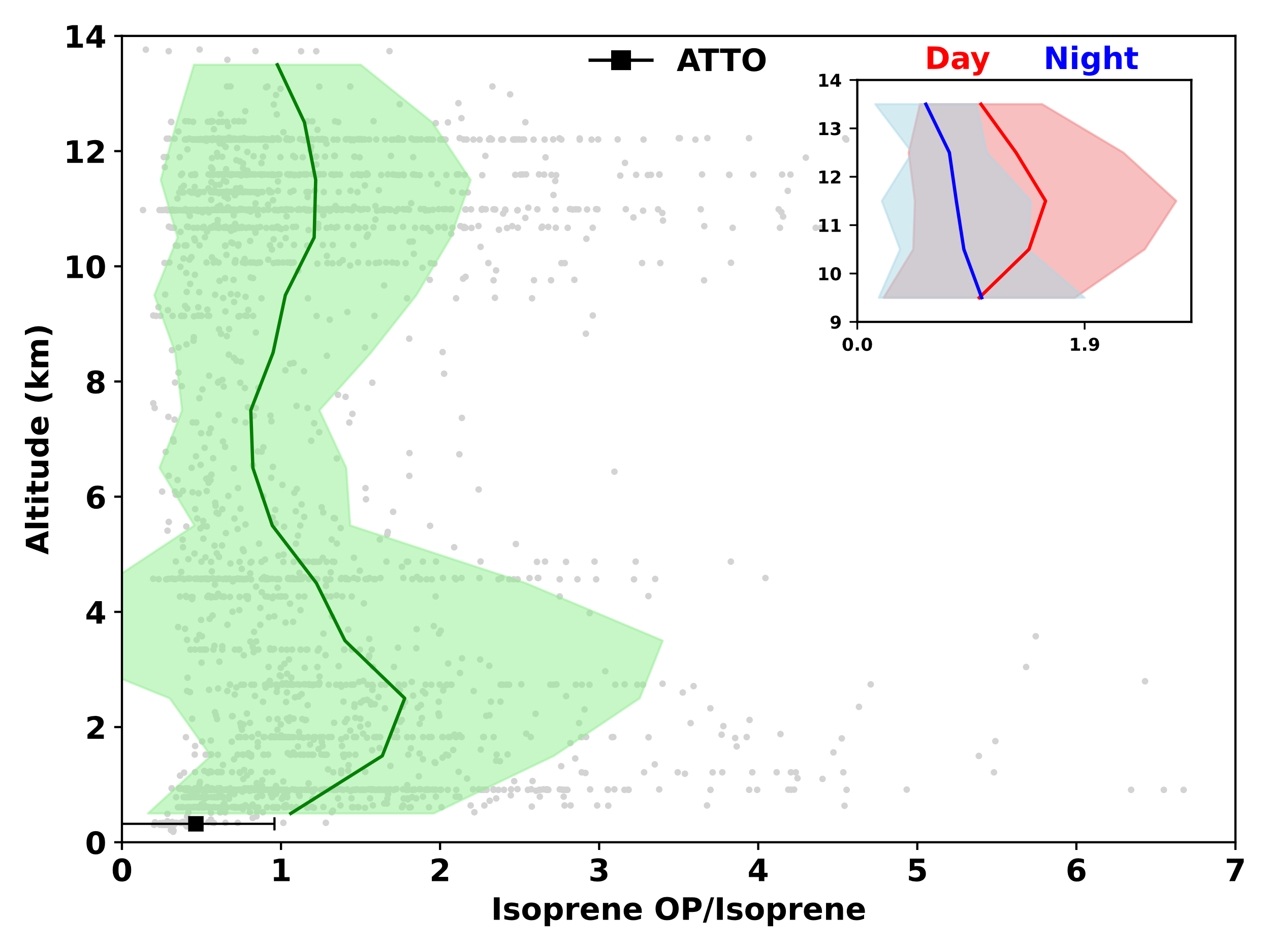
**

**Supplementary Figure 5.** Vertical profiles of the ratio of isoprene oxidation products (OP)/isoprene during the CAFE Brazil campaign. Gray dots are 1 min data, while mean values are represented by solid green line (1 km bin), the shaded light-green area shows the standard deviation and black square with error bars represents the mean value of Amazon Tall Tower Observatory (ATTO, 320 m) data measured during flight day. The inset plots show the mean vertical profiles of Isoprene OP/Isoprene at higher altitudes (9-14 km) during day and nighttime. Only data points above the limit of detection (LOD) of isoprene and isoprene OP are used for this plot.


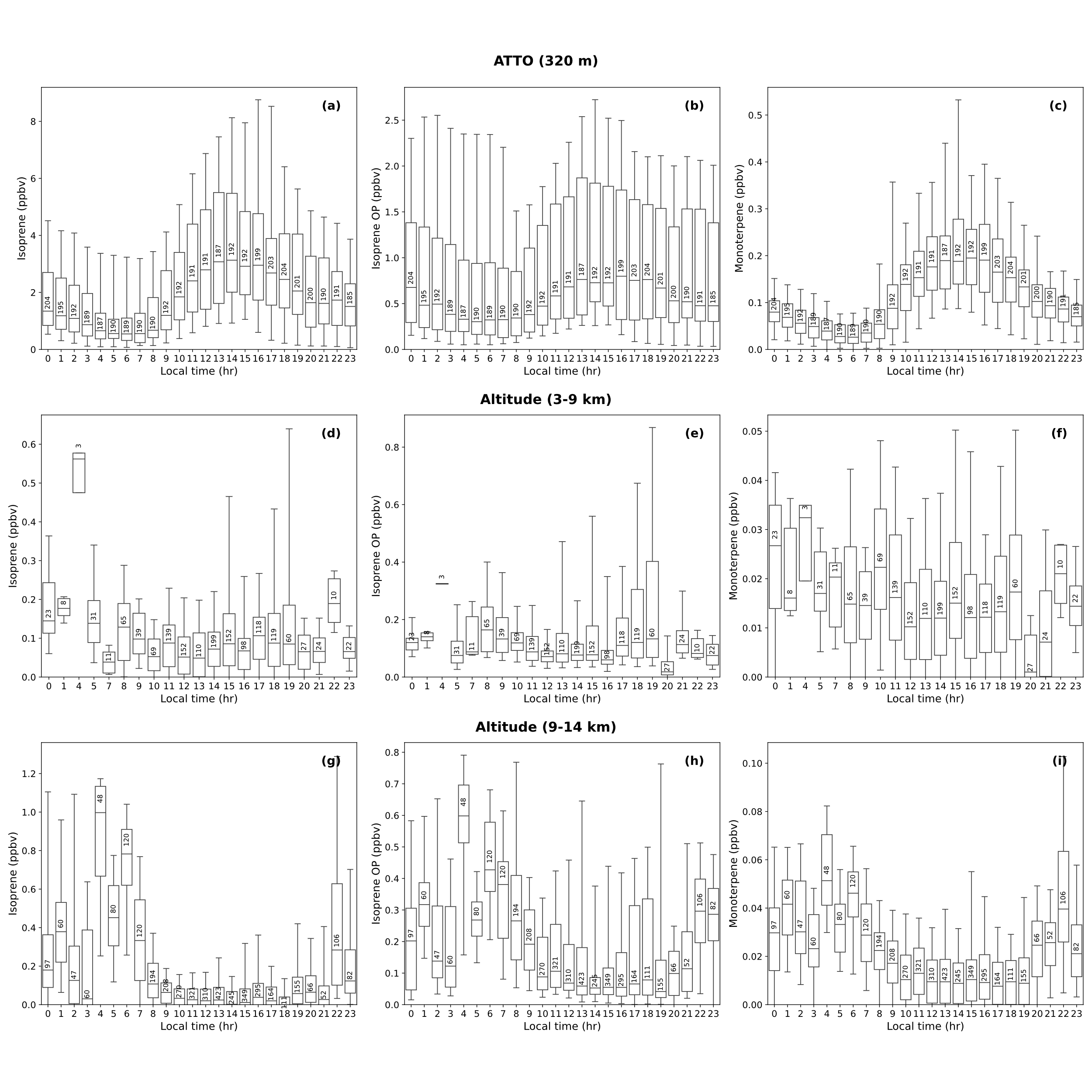


**Supplementary Figure 6.** Diurnal variations (24-hour) of isoprene, isoprene oxidation products (OP) and monoterpenes mixing ratios at different altitude over the amazon rainforest. The box-whisker plot represents the median, lower quartile, upper quartile, and the 5th and 95th percentiles for 1-hour bins measured at Amazon Tall Tower Observatory (ATTO, 320 m, a-c), the middle troposphere (3 to 9 km, d-f), and the upper troposphere (9 to 14 km, g-i). Panels a, d, and g correspond to isoprene; b, e, and h to its oxidation products; and c, f, and i to monoterpenes. The whiskers include all data points. The numbers within the boxes indicate the number of data points used to generate the box and whisker plots.

**
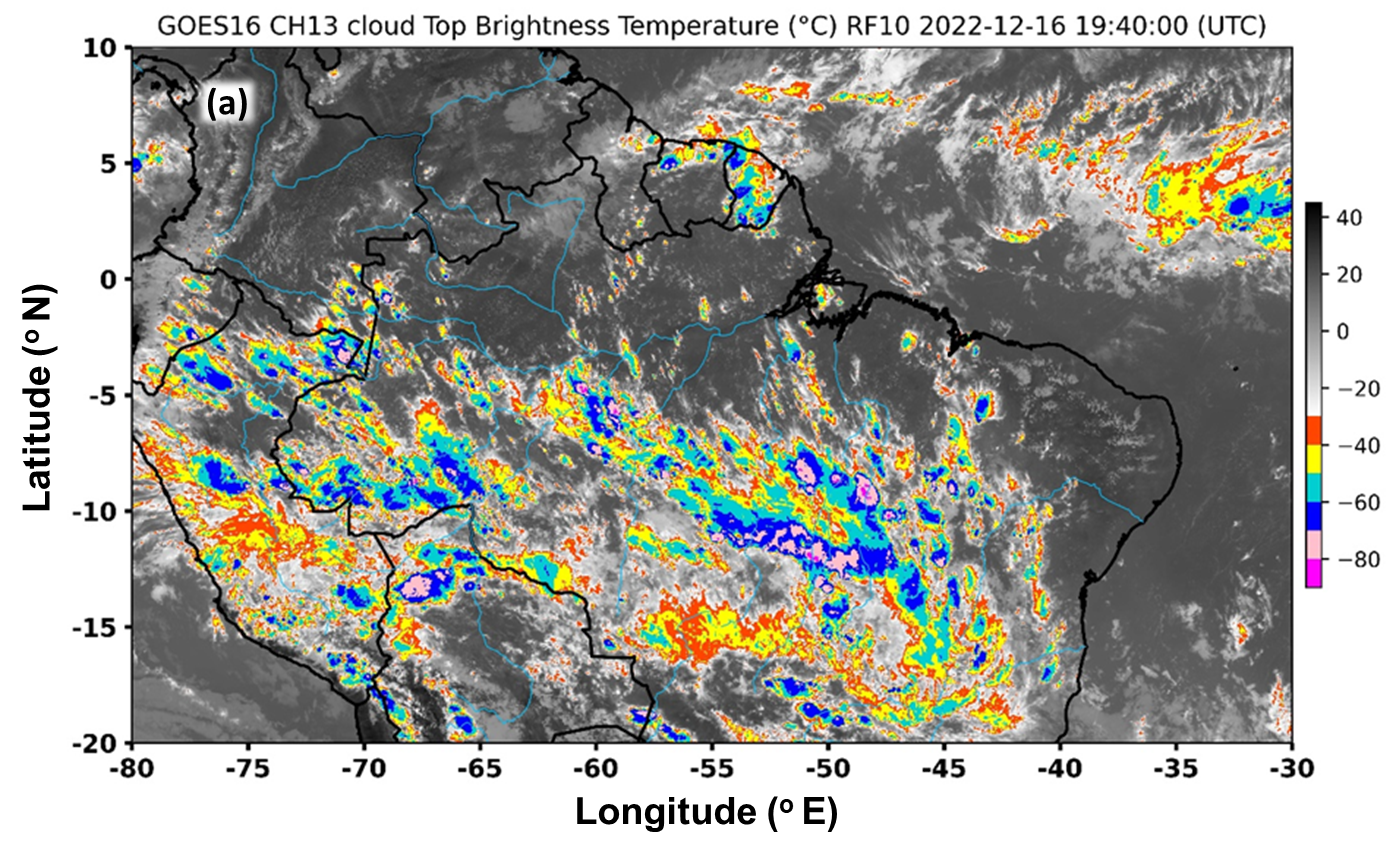
**

**
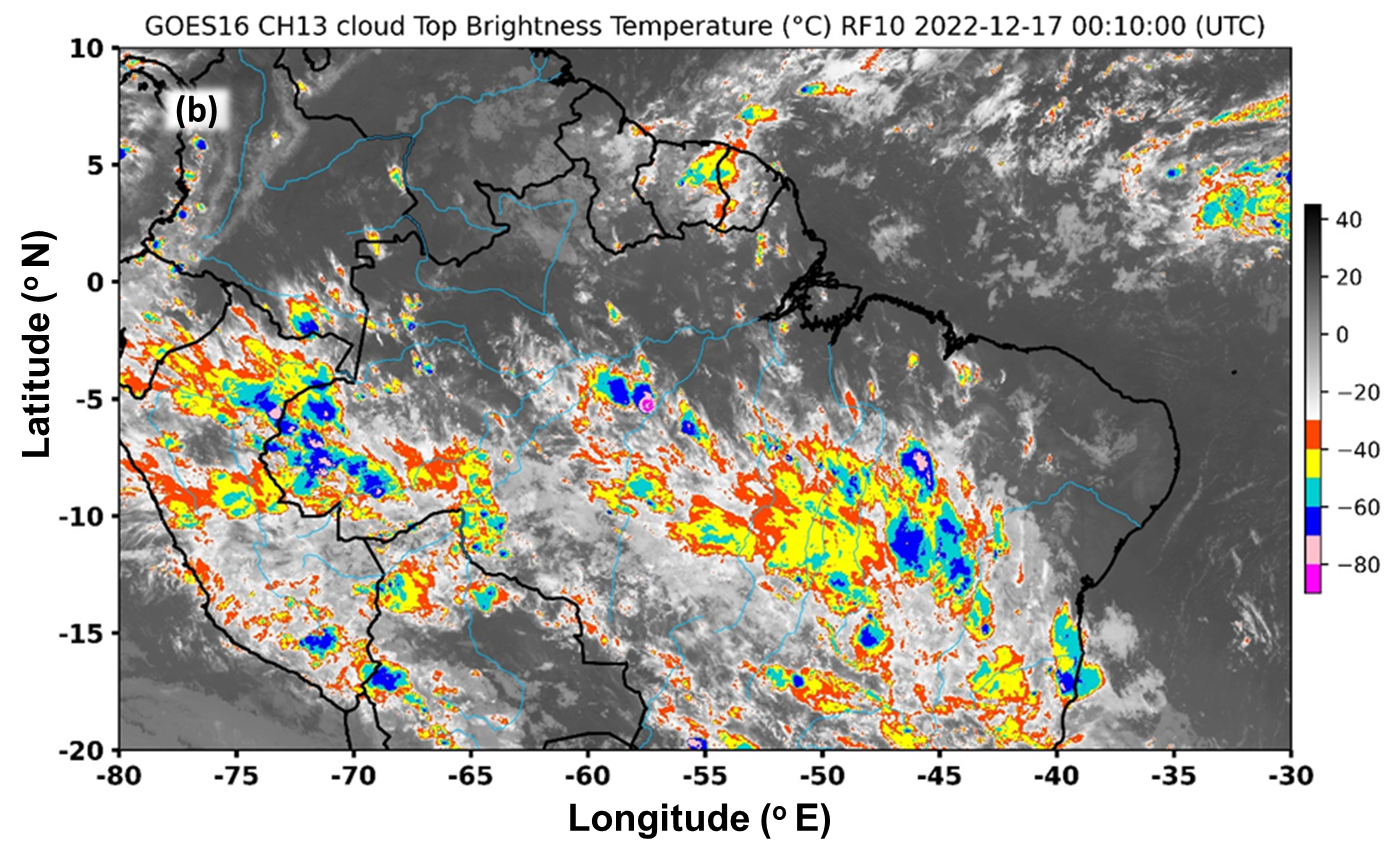
**

**Supplementary Figure 7.** The cloud-top brightness temperature for flight 10. The color bar represents the cloud brightness temperature (°C). The deep convective systems during a) daytime and b) nighttime for flight 10 are shown. Infrared satellite image (GOES-16, band 13: 10.3 µm; <https://ftp.cptec.inpe.br/goes/goes16/retangular/ch13/2022/12/>) indicating the approximate cloud-top brightness temperatures at 19:40 (15:40 local time) and 00:10 UTC (20:10 local time). Temperatures below −40 °C are colored. The map in the figures were made with Natural Earth. Free vector and raster map data @ naturalearthdata.com

**
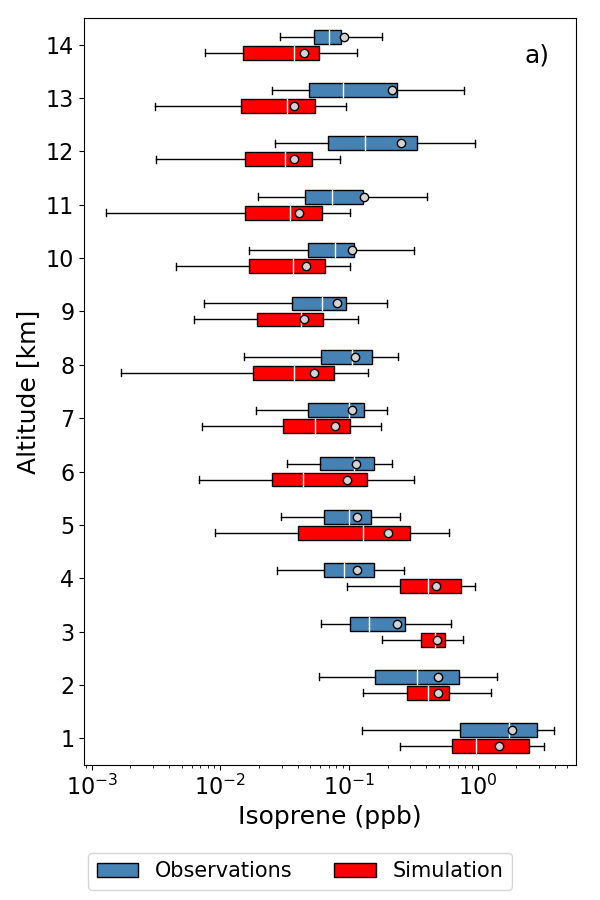

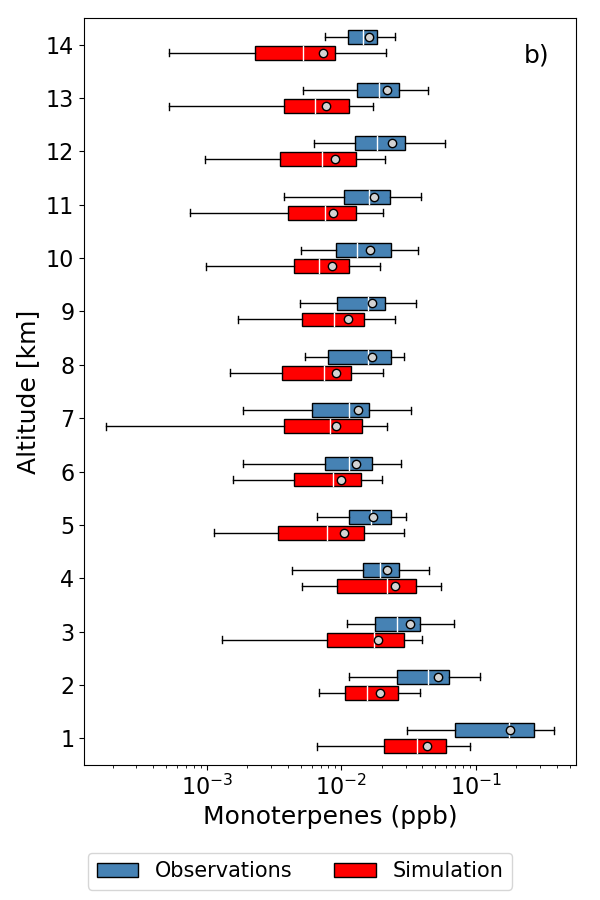
**

**Supplementary Figure 8.** Comparison of the vertical profile of observed and simulated a) isoprene and b) monoterpenes mixing ratios during the CAFE-Brazil campaign (December 2022 to January 2023). The box-whisker plot represents the mean (circle), median, lower quartile, upper quartile and the 5th and 95th percentiles for 1km bins. In the free and upper troposphere, the simulated mixing ratios mostly fall below the detection limit. To facilitate comparison with the observations, the simulated values were adjusted ("smeared") using instrument noise. The observation and model data are represented by blue and red color respectively.


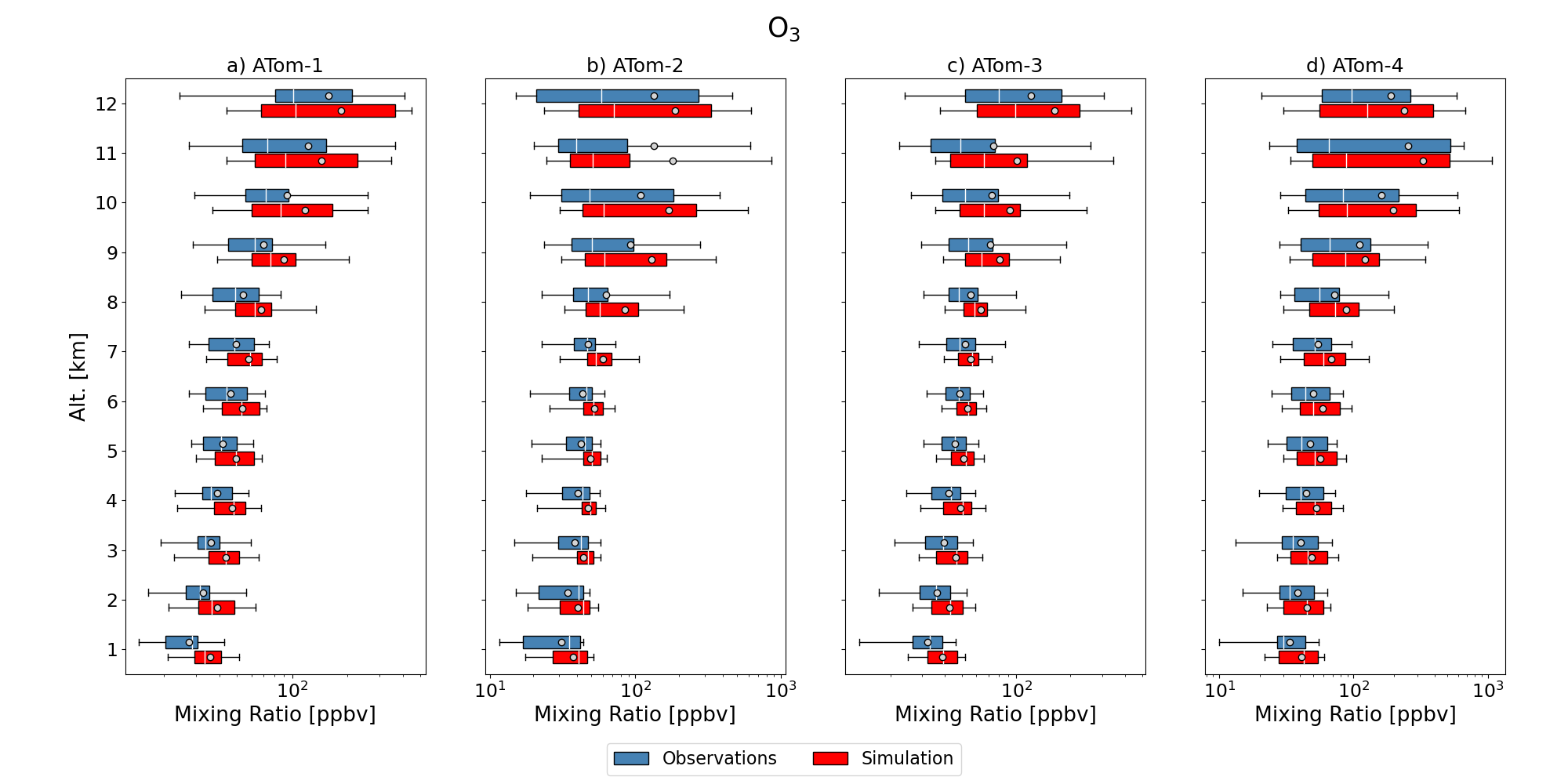
**Supplementary Figure 9**. Comparison of vertical profiles of observed and simulated O_3_ mixing ratios during the NASA Atmospheric Tomography (ATom) mission in the four sub-campaigns at different seasons: a) ATom-1 (July/August 2016), b) ATom-2 (January/February 2017), c) ATom-3 (September/October 2017), and d) ATom-4 (April/May 2018). The box-whisker plot represents the mean (circle), median, lower quartile, upper quartile and the 5th and 95th percentiles for 1km bins. The observation and model data are represented by blue and red color respectively. Observations are taken from Elkins et al. (2020)^1^.


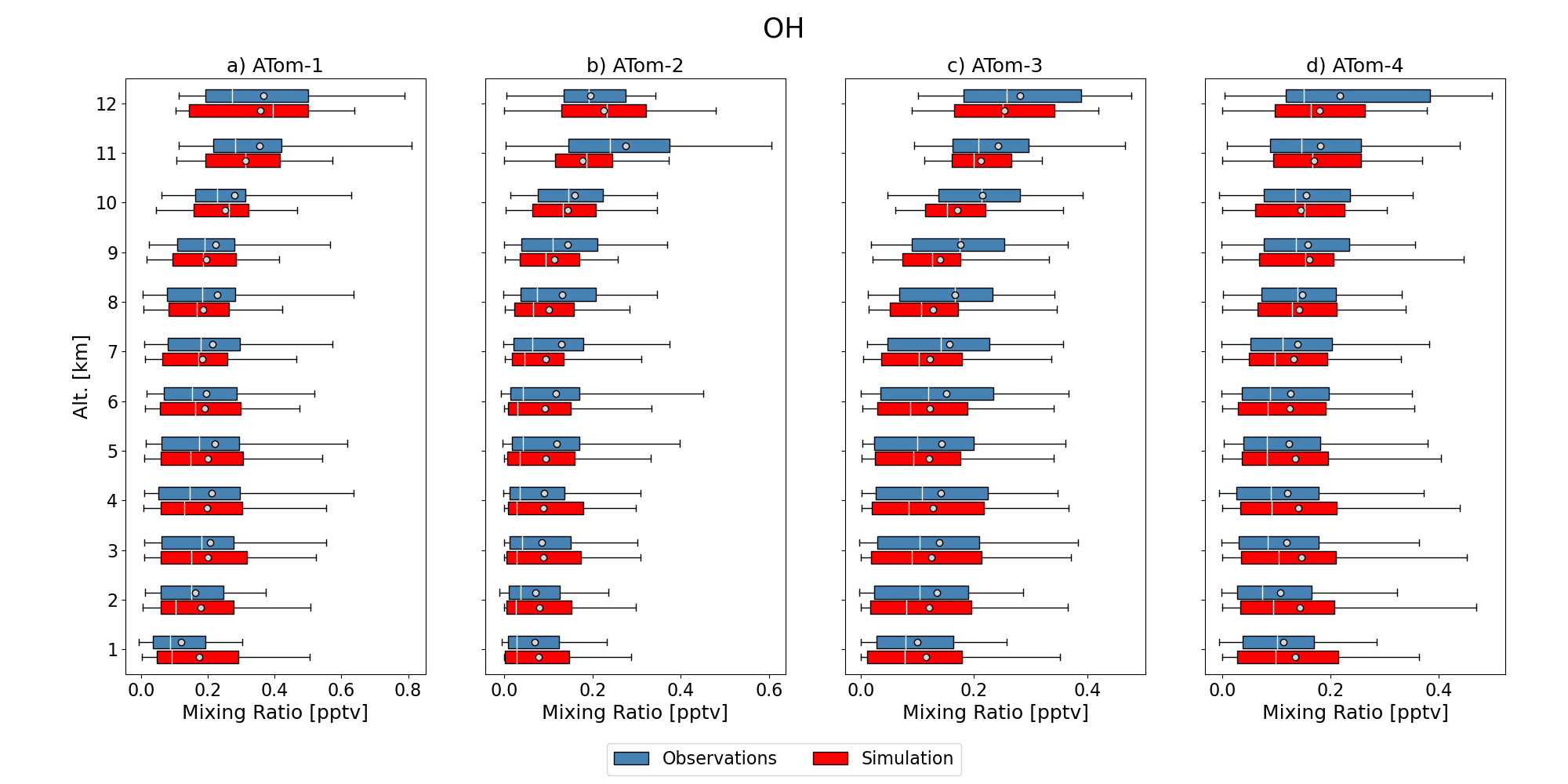


**Supplementary Figure 10**. Comparison of vertical profiles of observed and simulated OH mixing ratios during the NASA Atmospheric Tomography (ATom) mission in the four sub-campaigns at different seasons: a) ATom-1 (July/August 2016), b) ATom-2 (January/February 2017), c) ATom-3 (September/October 2017), and d) ATom-4 (April/May 2018). The box-whisker plot represents the mean (circle), median, lower quartile, upper quartile and the 5th and 95th percentiles for 1km bins. The observation and model data are represented by blue and red color respectively. Observations are taken from Brune et al. (2021)^2^.


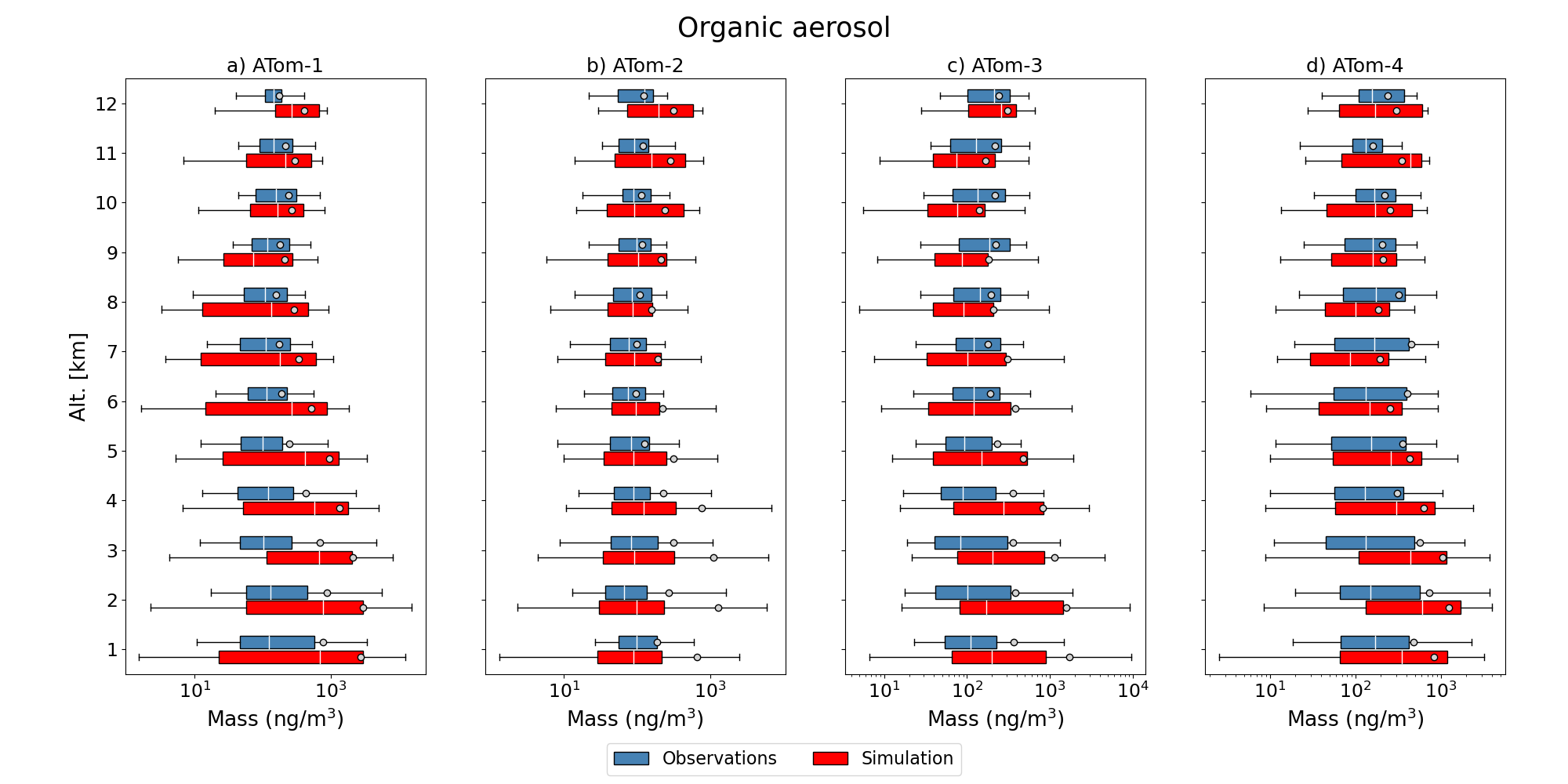


**Supplementary Figure 11.** Comparison of vertical profiles of observed and simulated organic aerosol mass concentrations during the NASA Atmospheric Tomography (ATom) mission in the four sub-campaigns at different seasons: a) ATom-1 (July/August 2016), b) ATom-2 (January/February 2017), c) ATom-3 (September/October 2017), and d) ATom-4 (April/May 2018). The box-whisker plot represents the mean (circle), median, lower quartile, upper quartile and the 5th and 95th percentiles for 1km bins. The observation and model data are represented by blue and red color respectively. Observations are taken from Jimenez et al. (2019)^3^.


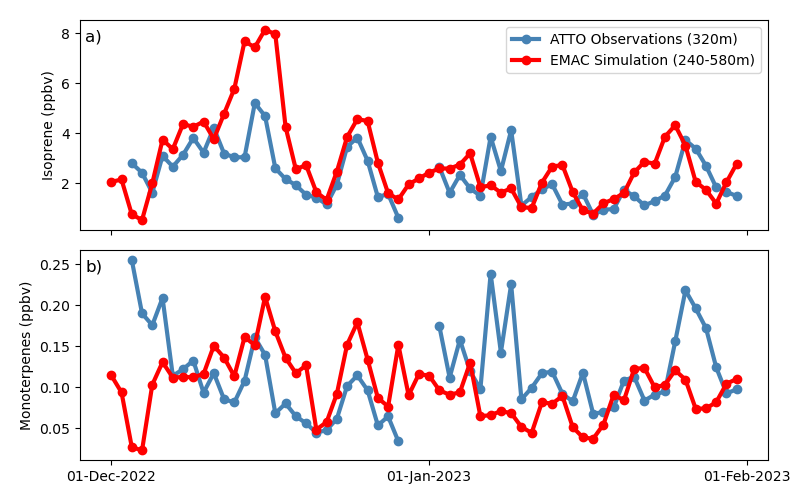


**Supplementary Figure 12**. Comparison of the time series of modeled and observed (a) isoprene and (b) monoterpene mixing ratios at the Amazon Tall Tower Observatory (ATTO, 320 m), and model simulated values. The blues line shows observations, while the red line shows the model output extracted from the grid cell covering 240 to 580 m.

**
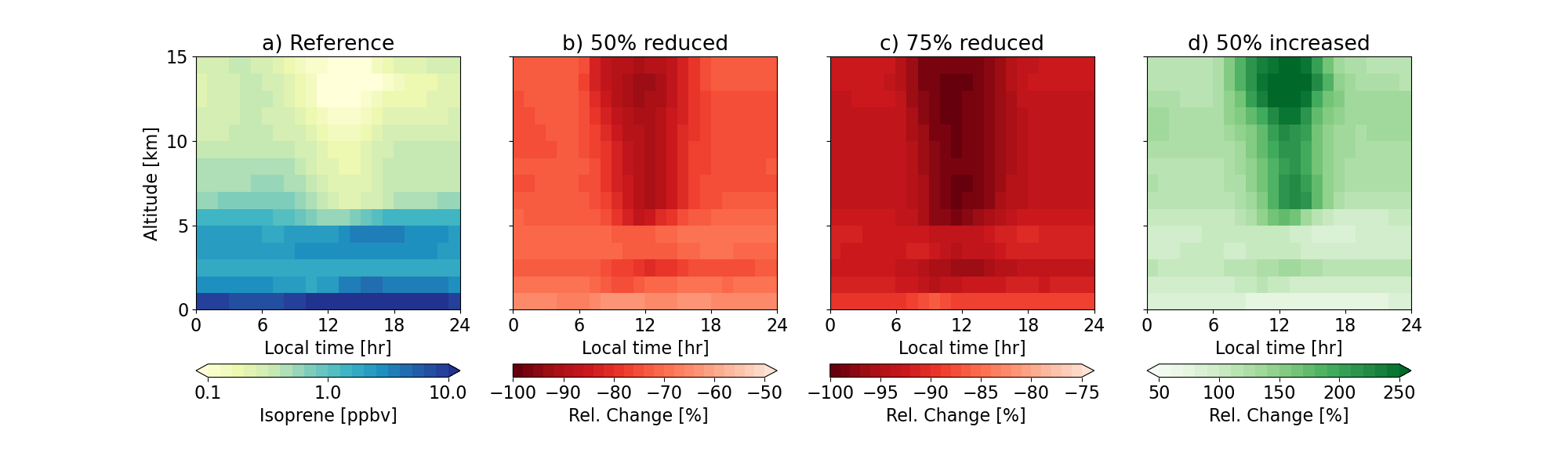
Supplementary Figure 13.** a) Simulated average diel cycle of maximum isoprene mixing ratios between December 2022 and January 2023 over the Amazon basin from the surface to the upper troposphere (UT). The diurnal profiles of relative changes in isoprene under simulations with b) 50%, c) 75% reduction and d) a 50 % increase in the biogenic volatile organic compounds (BVOCs) emission. Highest boundary layer mixing ratios occur in the afternoon, and the highest UT isoprene mixing ratios in the early morning. The sensitivity simulations show a non-linear decrease (increase) in the UT, especially in the daytime, in response to decreased (increased) BVOC emissions.


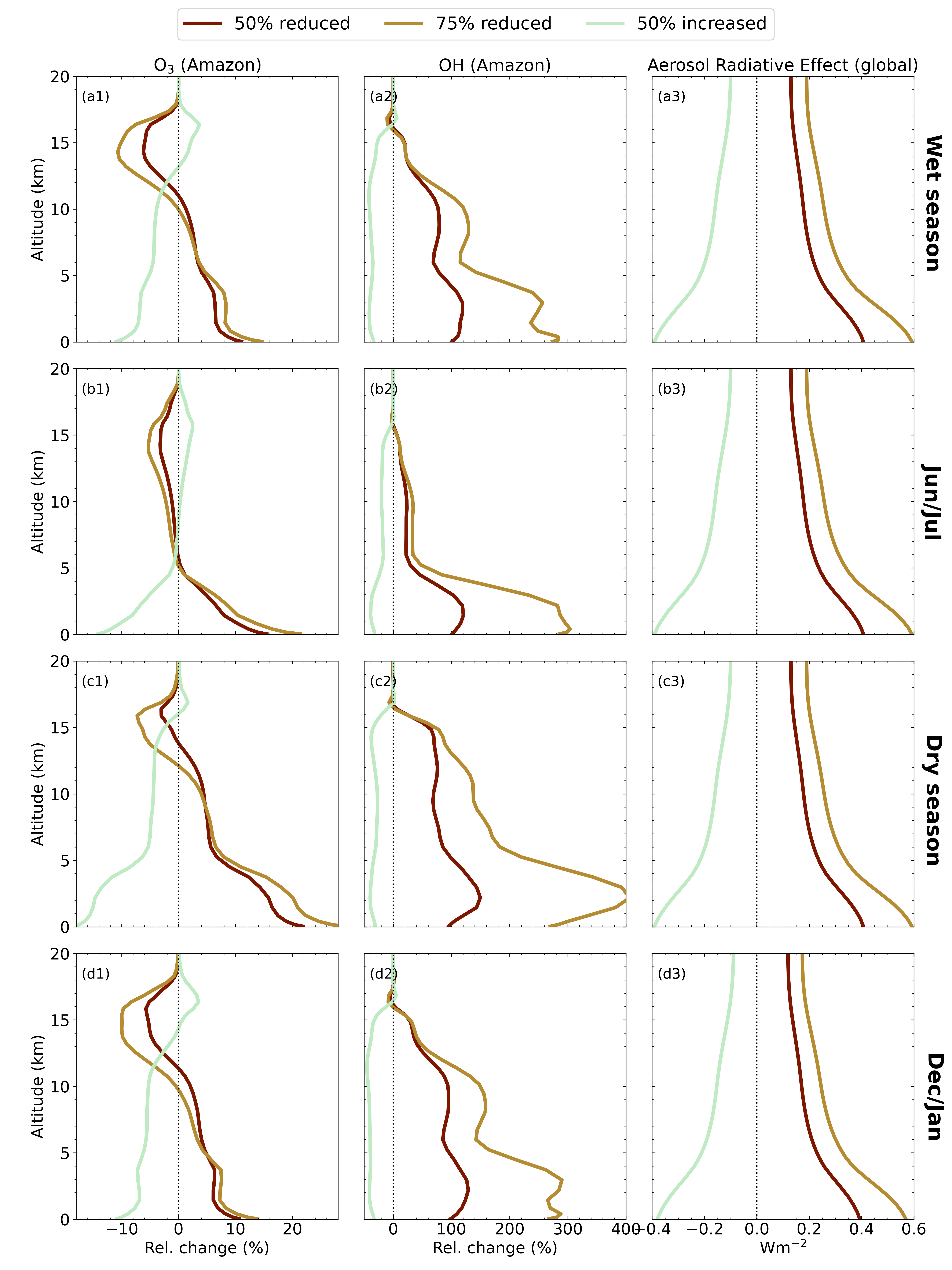
**Supplementary Figure 14**. Seasonal analysis of the relative changes in ozone and OH, along with the global direct aerosol radiative effect for the three sensitivity studies, analogously to Figure 5. The panel labels represent: (a1–a3) wet season, (b1–b3) June/July, (c1–c2) dry season, and (d1–d3) December/January. The wet season is defined from February to May and the dry season from August to November, according to Andreae et al. (2015)^4^.


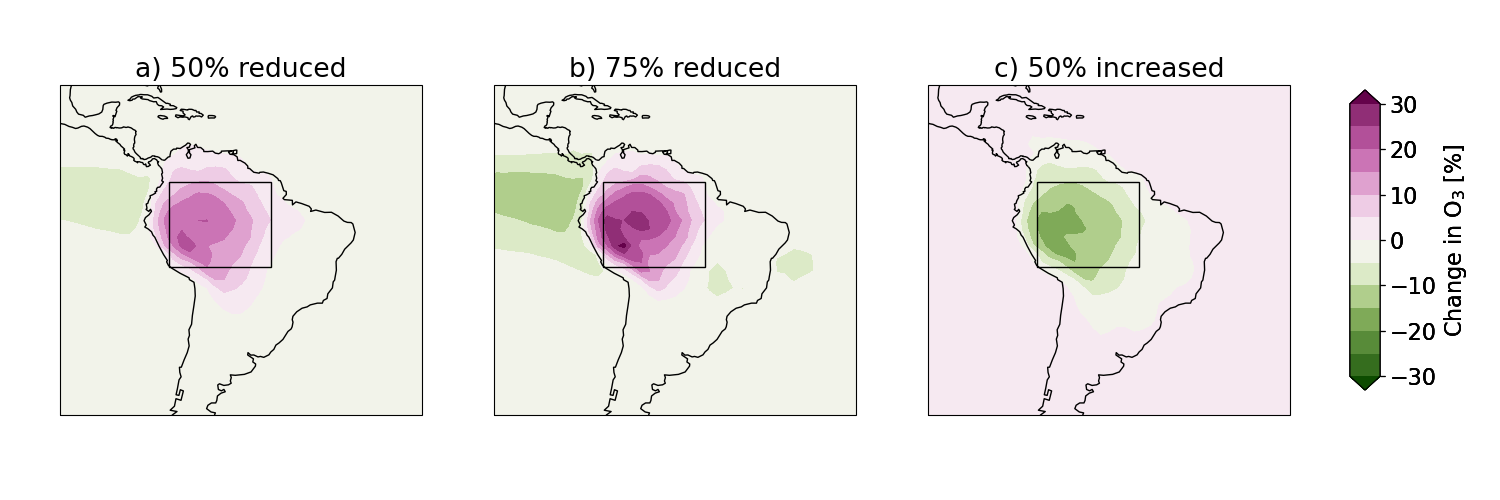
**Supplementary Figure 15**. The relative change in ozone after reducing a) 50 and b) 75 % and c) increasing 50% of biogenic volatile organic compounds (BVOCs) emission in the boundary layer of Amazon rainforests. The square shows the area referred to as the Amazon basin in simulations. The map in this figure was made with Natural Earth. Free vector and raster map data @ naturalearthdata.com.


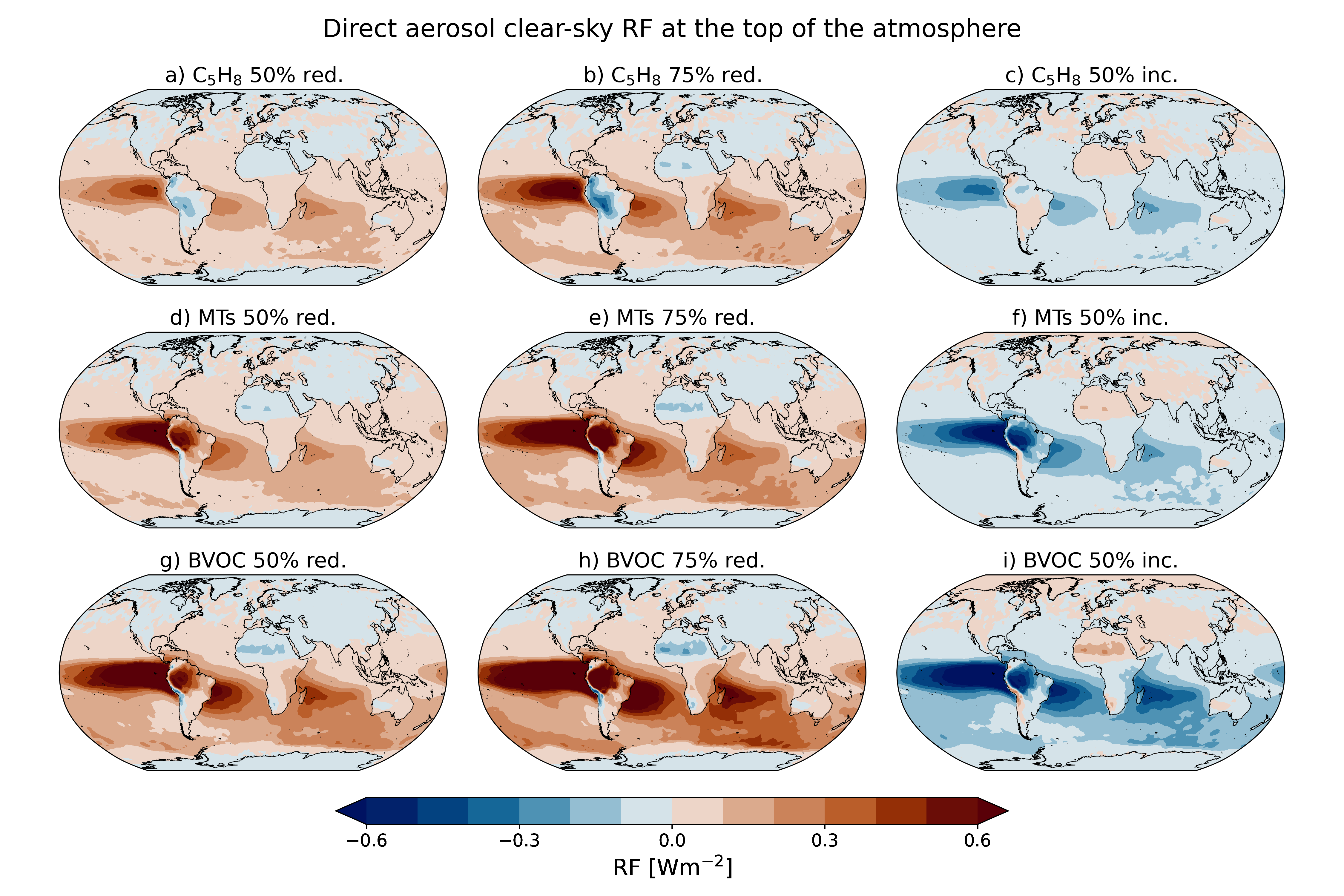


**Supplementary Figure 16.** Direct aerosol clear sky radiative forcing (RF) at the top of the atmosphere due to changes in aerosol concentration by reducing biogenic volatile organic compounds (BVOCs): a), d), and g) represent a 50% reduction (red.) in isoprene, monoterpenes, and total BVOCs, respectively; b), e), and h) depict a 75% reduction (red.) in isoprene, monoterpenes, and total BVOCs, respectively, over the Amazon Forest; c), f), and i) show a 50% increase (inc.) in isoprene, monoterpenes, and total BVOCs, respectively. The map in this figure was made with Natural Earth. Free vector and raster map data @ naturalearthdata.com


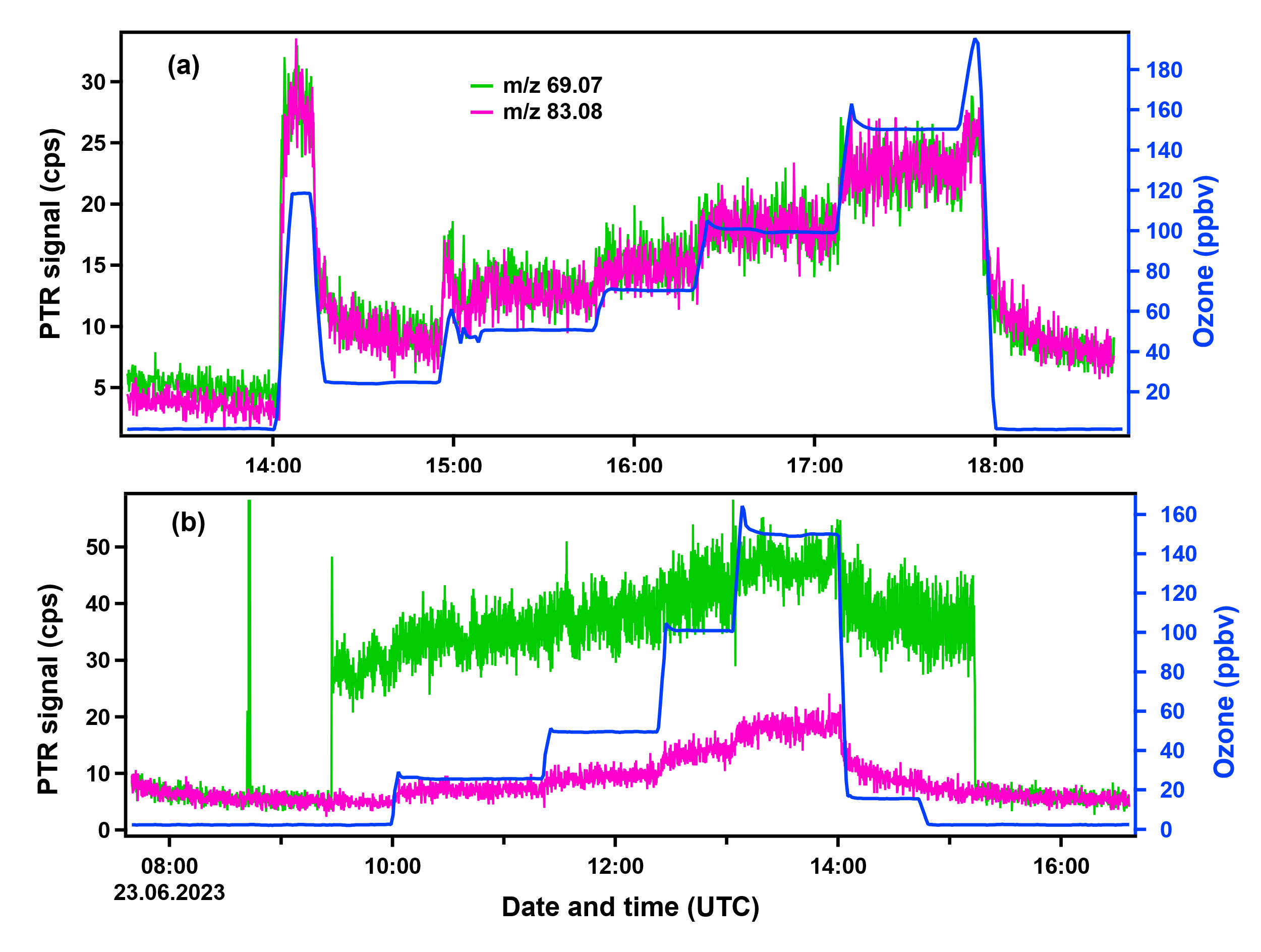
  **Supplementary Figure 17.** Time series of the signal at m/z 69.07 (C_5_H_8_H+), the tracer m/z due to ozone interference (m/z 83.08) and ozone measured during the lab experiment. a) without inserting isoprene and b) with isoprene during the experiment.


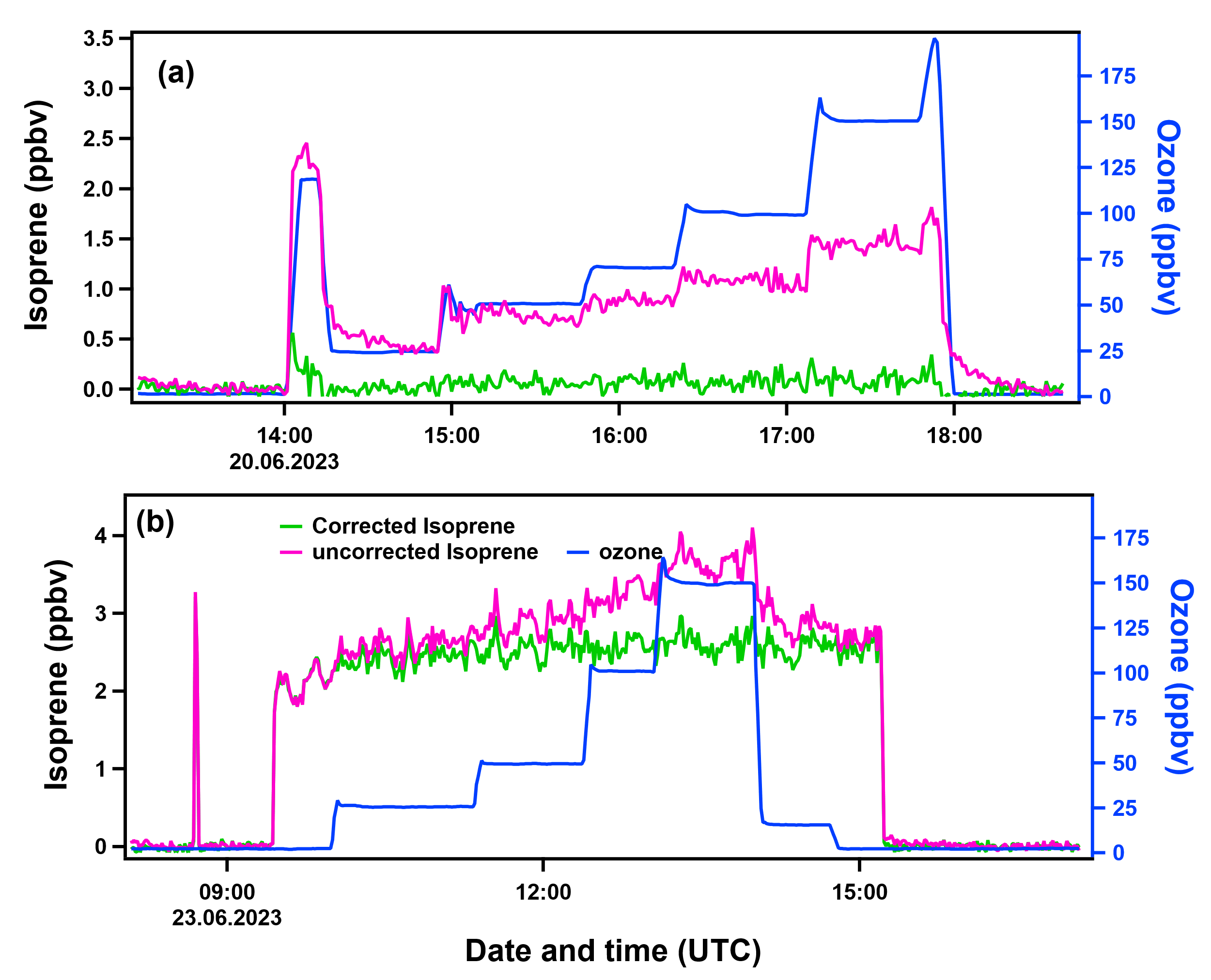


**Supplementary Figure 18.** Isoprene correction with ozone. Time series of isoprene measured by PTR-TOF-MS (uncorrected), after the correction and ozone during the laboratory experiment. a) without inserting isoprene and b) with isoprene during the experiment.


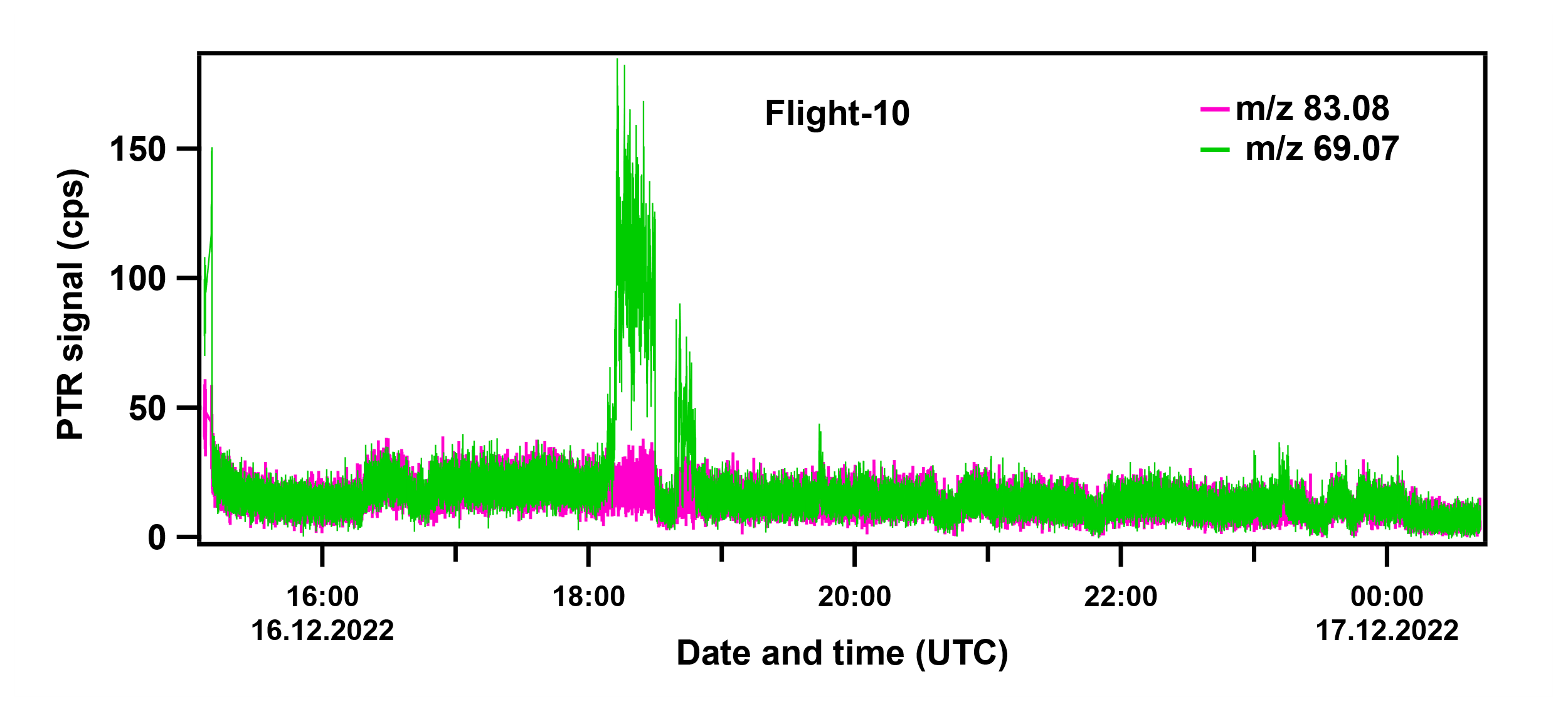
**Supplementary Figure 19.** Time series of the signal at m/z 69.07 (C_5_H_8_H+) and the tracer m/z 83.08 due to ozone interference measured for Flight-10 during the CAFE-Brazil campaign.


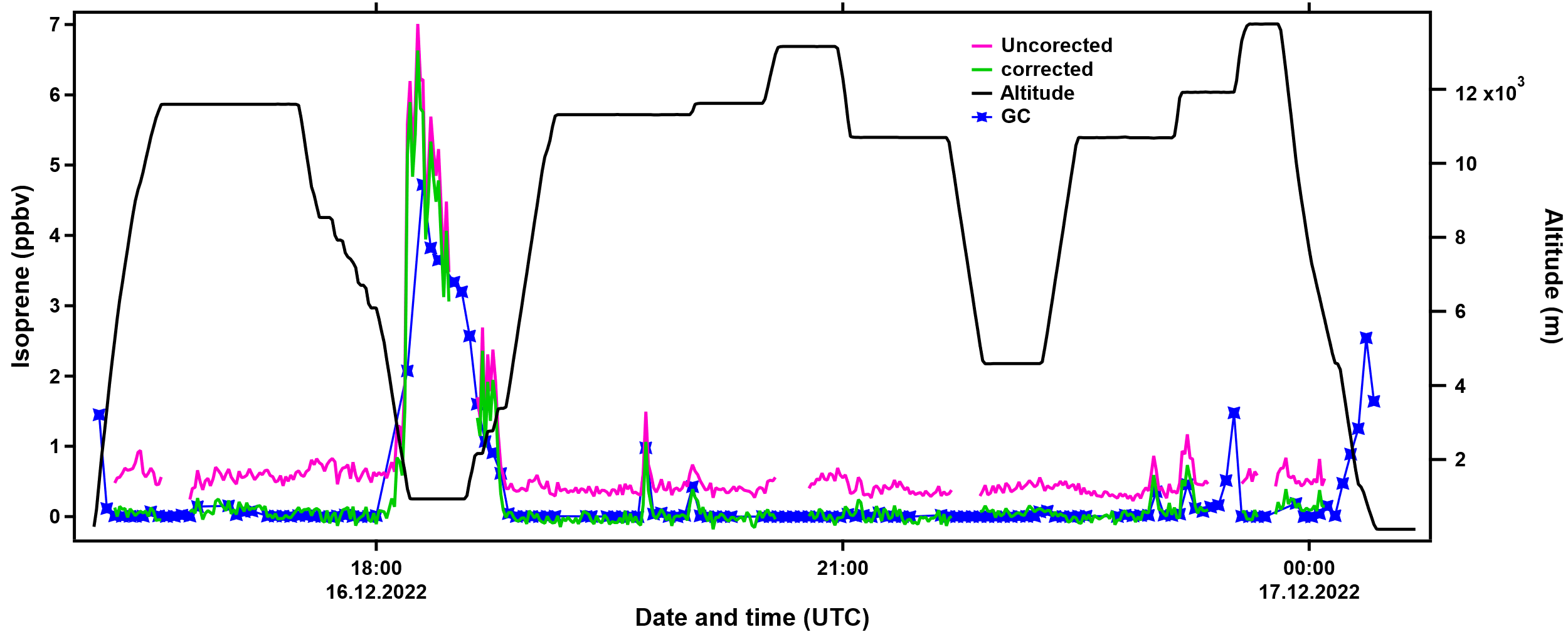


**Supplementary Figure 20.** Time series of corrected isoprene for flight 10. Time series of isoprene measured by identical Proton-Transfer-Reaction Time Of Flight Mass Spectrometers (PTR-TOF-MS) (uncorrected), after the correction and measured by Gas chromatography-mass spectrometer (GC-MS) during the CAFE-Brazil campaign in December 2022 to January 2023 over the Amazon rainforest.


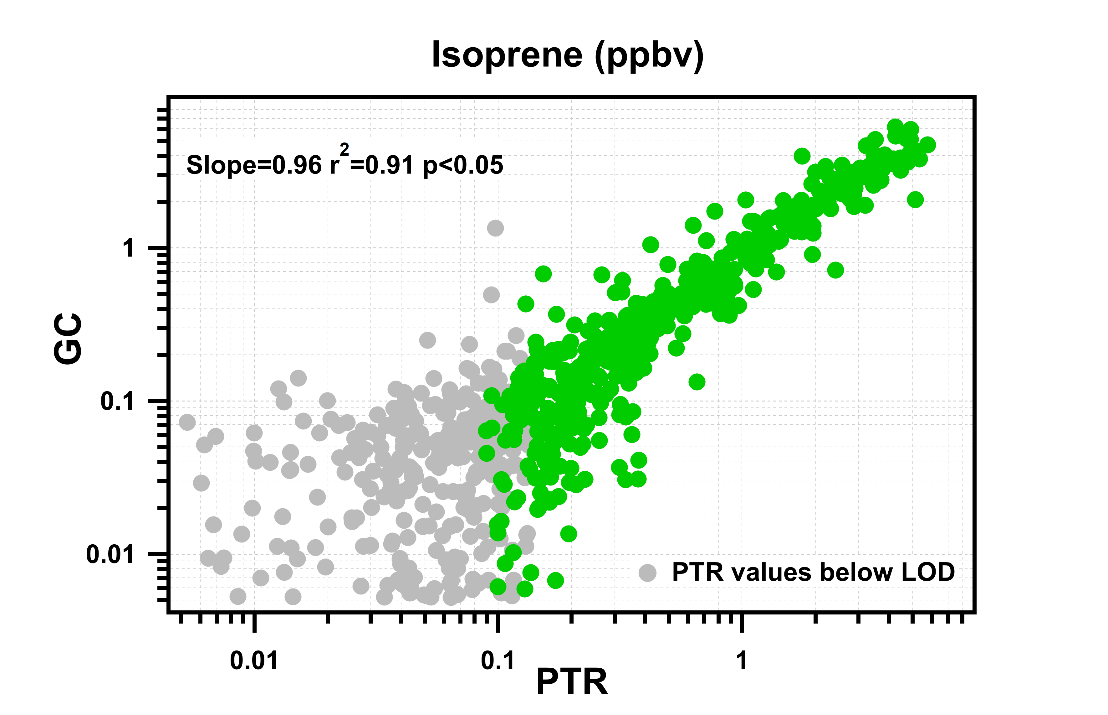


**Supplementary Figure 21.** Correlation plot of isoprene measured by Proton-Transfer-Reaction Time Of Flight Mass Spectrometers (PTR-TOF-MS) after the correction stated in the Supplementary Text 1 and the data from the fast Gas chromatography-mass spectrometer (GC-MS) during the CAFE-Brazil campaign in December 2022 to January 2023 over the Amazon rainforest. The slope and r² values shown in the graph are calculated using a linear regression fit.

**Supplementary Text 1**

***Isoprene Correction-***

On first analysis the mixing ratios (MRs) of isoprene measured using Proton-Transfer-Reaction Time Of Flight Mass Spectrometers (PTR-TOF-MS) did not always agree with the Gas chromatography-mass spectrometer (GC-MS) data during the campaign. Several studies have reported the fragmentation of certain aldehydes at the same mass of isoprene (m/z 69.069) using PTR-TOF-MS due to the influence of ozone^5,6^. During the CAFE Brazil campaign, the PTR-TOF-MS measured higher isoprene than the GC-MS at higher altitudes with higher ozone concentrations, leading to elevated background values (~200 ppt) during all the flights. Laboratory experiments were therefore performed with the same experimental inlet setup as the aircraft to characterize the signal with the aim of determining a correction for the PTR-TOF-MS isoprene signal. The experimental setup is discussed in the study by Ernle et al., 2022^5^. Four conditions were considered to investigate the interference of ozone on m/z 69.069. The first condition involved no isoprene injection, with variation in ozone levels (0, 25, 50, 70, 100, 150, 170 ppb) using synthetic air and zero relative humidity (RH) (Supplementary Figure 17a). In the second condition, a fixed isoprene level was maintained, with different ozone levels (0, 25, 50, 70, 100, 150 ppb) at zero RH (Supplementary Figure 17b). The third condition maintained a constant RH (~25%) and zero isoprene, with varying ozone levels. Finally, the fourth condition involved fixed RH (~40%) and isoprene (~2.5 ppb), with variation in ozone levels. During the measurement, we observed that when the ozone was injected, the signal of m/z 69.069 started increasing without introducing any isoprene. At the same time, m/z 83.085 also increased proportionally to the isoprene signal (Supplementary Figure 17a). When isoprene was injected, the signal of isoprene increased while there was no change in the signal of m/z 83.085 observed (Supplementary Figure 17b). Therefore, m/z 83.085 was used as the tracer for the ozone interference at the isoprene mass and used to correct the isoprene signal. For the correction, the signal of m/z 83.085 is subtracted from m/z 69.069 in the raw data before the calibration factor was applied for the final calculation (Supplementary Figure 18a & b). We also observed the same influence on the level of isoprene during CAFE Brazil and applied this procedure for the correction of the field data (Supplementary Figure 19 & 20). After correction, the measured values of isoprene from both instruments show an excellent agreement (Supplementary Figure 20 & 21).

**Supplementary Text 2**

Method: GC-MS

The fast GC-MS comprises a customized Gas Chromatograph (GC) combined with a commercial quadrupole Mass Spectrometer (MS, Agilent Technologies 5973MSD). Ambient air is sampled at a flow rate of 200 sccm through the same Trace Gas Inlet (TGI, Enviscope GmbH) that the PTR-MS is connected. The air is transported to the instrument via a 2m heated Teflon line (0.635 cm diameter) equipped with a sodium thiosulfate ozone scrubber^6^. Further, the air is pre-concentrated in a liquid nitrogen-cooled cryo-concentrator, where it undergoes sequential drying at -10°C, enrichment of VOCs at -160°C for one minute, and final concentration within a small volume at -160°C before rapid heating for sample injection into the GC. Separation is performed using a DB-624 UI capillary column (10 m, 0.25 mm, 1.4 µm; Agilent Technologies). The GC oven follows a temperature program starting at 30 °C for 50 s, then increasing to 200 °C at a rate of 1.8 °C s^−1^, and maintaining 200 °C until the end of the chromatogram. After separation, the components are ionized at 70 eV and detected by MS in selected ion mode (SIM). The configuration utilized for the CAFE-Brazil campaign enabled quantification of over 35 compounds within the 2.4 min chromatogram, with an overall time resolution of 3 minutes. Calibration was conducted using a gravimetrically prepared multicomponent pressurized standard (Apel-Riemer Environmental, stated accuracy 5%), with calibrations performed before, during, and after each flight. Isoprene was quantified at m/z 67 with retention time of 0.7 min, a detection limit of 5 ppt, and an uncertainty of approximately 10%.

**Supplementary References**

1. Elkins, J. W., Hintsa, E. J. & Moore, F. L. ATom: Measurements from the UAS Chromatograph for Atmospheric Trace Species (UCATS). *ORNL DAAC* (2020).

2. Brune, W., Miller, D. & Thames, A. ATom: Measurements from Airborne Tropospheric Hydrogen Oxides Sensor (ATHOS), V2. ORNL DAAC, Oak Ridge, Tennessee, USA. (2021).

3. Jimenez, J. *et al.* ATom: L2 Measurements from CU High-Resolution Aerosol Mass Spectrometer (HR-AMS), ORNL DAAC, Oak Ridge, Tennessee, USA. (2019).

4. Andreae, M. O. *et al.* The Amazon Tall Tower Observatory (ATTO): overview of pilot measurements on ecosystem ecology, meteorology, trace gases, and aerosols. *Atmospheric Chemistry and Physics* **15**, 10723–10776 (2015).

5. Ernle, L., Ringsdorf, M. A. & Williams, J. Influence of ozone and humidity on PTR-MS and GC-MS VOC measurements with and without Na_2_S_2_O_3_ ozone scrubber. *Atmospheric Measurement Techniques Discussions* 1–22 (2022).

6. Ernle, L. *et al.* Assessment of aldehyde contributions to PTR-MS m/z 69.07 in indoor air measurements. *Environ. Sci.: Atmos.* (2023).
